# Acute pancreatitis following endoscopic hemostasis of a periampullary Dieulafoy lesion: successful rescue by endoscopic retrograde cholangiopancreatography

**DOI:** 10.1055/a-2740-3158

**Published:** 2025-12-03

**Authors:** Giulia Palumbo, Ivo Boškoski, Andrea Tringali, Alessandra Farchione, Samuele Mafucci Orlandini, Fabrizio Termite, Cristiano Spada

**Affiliations:** 1 686177 Digestive Endoscopy Unit, Fondazione Policlinico Universitario Agostino Gemelli, IRCCS, Rome, Italy; 2 18654 Department of Diagnostic Imaging, Oncological Radiotherapy and Hematology, Fondazione Policlinico Universitario Agostino Gemelli, IRCCS, Rome, Italy

Periampullary Dieulafoy lesions are a rare source of upper gastrointestinal bleeding. Endoscopic treatment in this region carries a risk of post-procedural pancreatitis due to inadvertent papillary obstruction 1 2 .

We present the case of an 87-year-old man with a history of recurrent gastrointestinal bleeding due to angiodysplasias and Dieulafoy lesions, who was admitted with melena and dyspnea. On presentation, he was hemodynamically stable but severely anemic (Hb 5.2 g/dL). Laboratory tests showed normal liver function and pancreatic enzyme levels. Following transfusion of three units of red blood cells, CT imaging identified a hypervascular lesion in the second portion of the duodenum ( Fig. 1 ). Urgent esophagogastroduodenoscopy revealed active bleeding from a periampullary Dieulafoy lesion, treated with epinephrine injection and placement of two endoscopic clips.

**Fig. 1:**
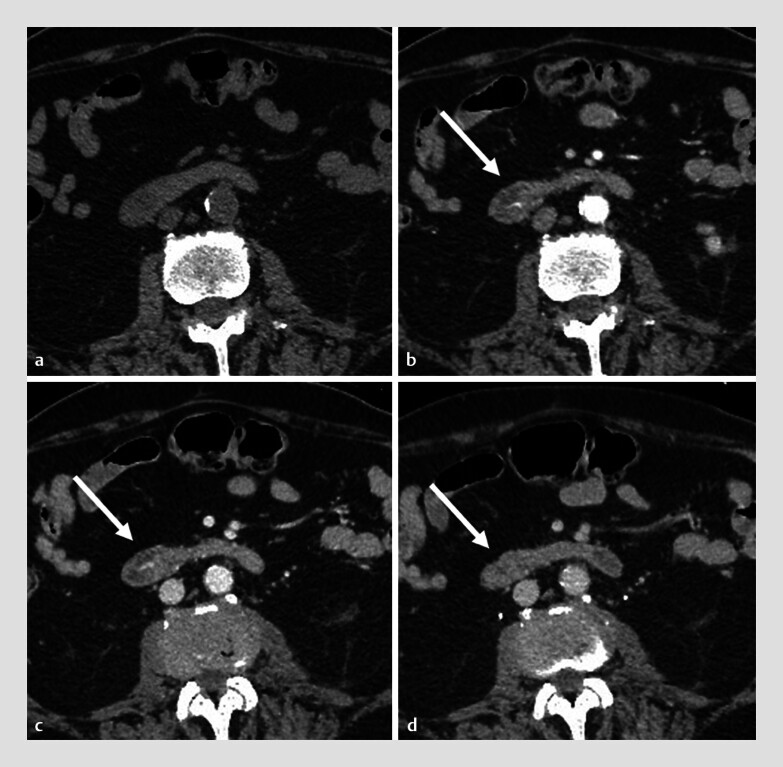
Unenhanced ( **a** ) and contrast-enhnaced multidetector-CT ( **b, d** ), showing in the descending duodenum progressive contrast medium extravasation on vascular lesion during dynamic arterial ( **b** ), portal venous ( **c** ) and equilibrium ( **d** ) phases.

Fourteen hours later, the patient developed mesogastric pain. Laboratory findings showed a marked elevation in amylase (1236 U/L) and a mild increase in bilirubin (1.3 mg/dL). CT scan reported duodenal wall hematoma and edematous acute pancreatitis. ERCP confirmed that the two endoclips were in place, but one directly over the papillary orifice ( Video 1 ). The obstructing clip was carefully removed using a foreign body retrieval forceps, resulting in immediate drainage of dense black bile. Both pancreatic and biliary ducts were successfully cannulated. A 7 Fr × 5 cm pancreatic stent was placed for ductal protection, followed by 6 mm hydrostatic dilation and deployment of a 10 Fr × 9 cm biliary stent for decompression.

Endoscopic removal of an endoclip obstructing the papilla, originally placed for hemostasis of a bleeding Dieulafoy. ERCP allowed cannulation of both biliary and main pancreatic ducts, followed by successful stenting and decompression.Video 1

Follow-up imaging 5 days later showed a reduction in pancreatic edema. Hemoglobin increased to 9.2 g/dL, and amylase levels decreased to 297 U/L.

This case illustrates a rare complication of clip-induced papillary obstruction and highlights the importance of early recognition and minimally traumatic clip removal using foreign body retrieval forceps followed by biliopancreatic stenting as an effective endoscopic strategy.

Endoscopy_UCTN_Code_TTT_1AR_2AK

## Notes

Endoscopy E-Videos https://eref.thieme.de/e-videos
*E-Videos* is an open access online section of the journal *Endoscopy* , reporting on interesting cases and new techniques in gastroenterological endoscopy. All papers include a high-quality video and are published with a Creative Commons CC-BY license. Endoscopy E-Videos qualify for HINARI discounts and waivers and eligibility is automatically checked during the submission process. We grant 100% waivers to articles whose corresponding authors are based in Group A countries and 50% waivers to those who are based in Group B countries as classified by Research4Life (see: https://www.research4life.org/access/eligibility/ ). This section has its own submission website at https://mc.manuscriptcentral.com/e-videos .
